# *Notes from the Field*: Adverse Events Following a Mass Yellow Fever Immunization Campaign — Kongo Central Province, Democratic Republic of the Congo, September 2016

**DOI:** 10.15585/mmwr.mm6612a7

**Published:** 2017-03-31

**Authors:** John O. Otshudiema, Nestor G. Ndakala, Maurice L. Loko, Elande-taty K. Mawanda, Gaston P. Tshapenda, Jacques M. Kimfuta, Abdou S. Gueye, Jacob Dee, Rossanne M. Philen, Coralie Giese, Christopher S. Murrill, Ray R. Arthur, Benoit I. Kebela

**Affiliations:** ^1^Epidemic Intelligence Service Program, CDC; ^2^Meningitis and Vaccine Preventable Diseases Branch, Division of Bacterial Diseases, National Center for Immunization and Respiratory Diseases, CDC; ^3^Field Epidemiology and Laboratory Training Program, Kinshasa, Democratic Republic of the Congo; ^4^Programme Elargi de Vaccination (Expanded Program on Immunization), Division Provinciale de la Santé, Kongo Central Province, Ministry of Health, Democratic Republic of the Congo; ^5^Division Provinciale de la Santé, Kongo Central Province, Ministry of Health, Democratic Republic of the Congo; ^6^Direction de Lutte Contre la Maladie, Ministry of Health, Kinshasa, Democratic Republic of the Congo; ^7^Division of Global Health Protection, Center for Global Health, CDC; ^8^Division of Global HIV and TB, Center for Global Health, CDC; ^9^Global Immunization Division, Center for Global Health, CDC.

On April 23, 2016, the Democratic Republic of the Congo (DRC) Ministry of Health reported an outbreak of yellow fever. As of May 24, 2016, among 41 confirmed yellow fever cases, 31 (75.6%) had occurred in Kongo Central Province, in the western part of the country bordering Angola (*1*), where a large yellow outbreak had begun in December 2015. In response, during May 25–June 7, 2016, the DRC Ministry of Health administered approximately 240,000 doses of yellow fever vaccine to all persons aged ≥9 months during a mass vaccination campaign in Matadi, one of 31 health zones in the Kongo Central Province. The administrative vaccination coverage (i.e., the number of vaccine doses administered divided by the most recent census estimates for the target population), was estimated to have reached >99%.

During the campaign, health workers in the Matadi Health Zone were trained to identify adverse events following immunization (AEFIs), complete case report forms, and send forms weekly to both provincial officials and a national expert committee for vaccine pharmacovigilance. Although a provisional classification of AEFIs by severity is made at peripheral and provincial levels at the time of an initial investigation, responsibilities at the national level are to guide the investigation of suspected serious AEFIs, classify them according to standard AEFI cause–specific definitions, recommend additional testing of biologic specimens if warranted, and determine causality.

Because identification of AEFIs through passive surveillance is limited by low reporting rates in Kongo Central Province (estimated <50%), active surveillance (review of hospital records and interviews with health care personnel) for AEFIs after receipt of yellow fever vaccine was piloted in the Matadi Health Zone after the campaign. Results obtained through active surveillance were compared with the results from the existing routine passive AEFI reporting system integrated into the Expanded Program on Immunization (EPI), which was established in the late 1970s to ensure that infants/children and mothers have access to routinely recommended vaccines.

An AEFI was defined for both the passive and active surveillance programs as any untoward medical occurrence (reported by either the vaccine recipient or a health worker) that occurs after immunization (≤30 days after the receipt of yellow fever vaccine) and which is not necessarily causally related to receipt of the vaccine. An active retrospective search to identify AEFIs was conducted at two referral health facilities in the Matadi Health Zone. Data were collected using the national EPI AEFI case investigation form, which was revised based on recommendations in the World Health Organization’s field guide, *Surveillance of Adverse Events Following Immunization Against Yellow Fever *(*2*). Trained EPI health zone and provincial surveillance supervisors identified potential AEFI cases through review of hospital registries and medical charts and interviews with emergency department personnel and community health workers. At the peripheral and provincial levels, identified AEFIs were provisionally classified as serious or nonserious. Serious AEFIs included those resulting in death, hospitalization or prolongation of hospitalization, persistent or major disability/incapacity; those that were life-threatening; or those that represented a congenital anomaly/birth defect in an infant after vaccination of the mother during pregnancy. All other AEFIs were classified as nonserious (*3*).

AEFIs identified through this comprehensive review were compared with those detected through passive surveillance during the immunization campaign to assess the completeness and representativeness of passive surveillance data. Overall, 15 AEFIs were identified by active surveillance among approximately 2,800 patient records reviewed at the two targeted referral hospitals, including eight AEFIs previously reported during the immunization campaign (Table). Two AEFIs were classified as serious and 13 as nonserious. The serious AEFIs comprised a spontaneous abortion that occurred after inadvertent administration of yellow vaccine early during an unrecognized pregnancy and a nonspecific gastrointestinal syndrome, both resulting in prolonged hospitalizations. Nonserious AEFIs included cutaneous allergic reactions, itching, fever, and injection site erythema. The incidences were 6.2 per 100,000 vaccine doses administered for all identified AEFIs and 0.8 for serious AEFIs. The AEFI incidence rate using the previous passive EPI surveillance data was 3.3 per 100.000 vaccine doses administered. Previous studies in African settings have found an expected AEFI rate of 8.2 per 100,000 yellow fever vaccine doses administered for all reported AEFIs and 0.4 for any serious AEFI (*4*).

**TABLE:** Adverse events following immunization (AEFIs) after a mass yellow fever vaccination campaign, identified through active surveillance system — Matadi Health Zone, Kongo Central Province, Democratic Republic of the Congo, September 2016

Patient	Sex	Age (yrs)	Date reported	Vaccine receipt to onset (days)	Description of AEFI (other associated medical conditions)*	Provisional classification peripheral level	Outcome
1^†^	F	23	5/27/2016	2	Cutaneous allergic reaction, rash, itching	Nonserious	Recovered
2	M	16	5/28/2016	3	Unexplained fever	Nonserious	Recovered
3	M	25	5/28/2016	2	Gastrointestinal syndrome, vomiting, fever	Nonserious	Recovered
4^†^	M	59	5/30/2016	2	Cutaneous allergic reaction, rash, itching	Nonserious	Recovered
5^†^	F	36	5/30/2016	2	Injection-site pain and erythema, tiredness, muscle pain	Nonserious	Recovered
6	M	3	5/30/2016	3	Undetermined hematuria and tiredness	Nonserious	Recovered
7^†^	F	26	5/31/2016	2	Cutaneous allergic reaction, rash, itching	Nonserious	Recovered
8^†^	F	49	5/31/2016	2	Cutaneous allergic reaction, rash, itching	Nonserious	Recovered
9^†^	F	14	5/31/2016	2	Cutaneous allergic reaction, rash, itching, fever	Nonserious	Recovered
10^†^	F	45	5/31/2016	2	Cutaneous allergic reaction, rash, itching, allergic reaction on lips	Nonserious	Recovered
11^†^	M	29	6/1/2016	2	Cutaneous allergic reaction, rash, itching, injection site pain and erythema	Nonserious	Recovered
12	F	25	6/1/2016	3	Allergic reaction on lips	Nonserious	Recovered
13	F	7	6/2/2016	2	Gastrointestinal syndrome, muscle pain, injection-site pain and erythema (severe malaria and urinary tract infection)	Serious	Recovered after 7-day hospitalization
14	M	17	6/4/2016	2	Eye allergic reaction, conjunctivitis	Nonserious	Recovered
15	F	22	6/11/2016	5	Spontaneous abortion of an unrecognized early pregnancy (endometritis)	Serious	Recovered after 7-day hospitalization

**Abbreviations:** AEFI = adverse event following immunization; F = female; M = male.

* Descriptions based on the Expanded Program on Immunization AEFI investigation forms, which were revised based on recommendations in the World Health Organization field guide for surveillance of AEFIs following yellow fever vaccination.

^†^ Detected by the routine passive surveillance system.

This enhanced surveillance program found that the passive yellow fever AEFI system failed to identify half of all AEFIs that were identified through active surveillance, including all of the serious AEFIs. The national expert committee for vaccine pharmacovigilance will validate all AEFIs identified through this evaluation; however, discrepancies between AEFIs identified through this pilot active surveillance and through passive surveillance highlight the need for an organized and active data collection system to supplement the lack of sensitivity of passive AEFI detection during a mass immunization campaign (*2*,*3*).
